# Longitudinal changes in habitual physical activity in adult people with cystic fibrosis in the presence or absence of treatment with elexacaftor/tezacaftor/ivacaftor

**DOI:** 10.3389/fspor.2024.1284878

**Published:** 2024-02-23

**Authors:** Wolfgang Gruber, Florian Stehling, Christopher Blosch, Stefanie Dillenhoefer, Margarete Olivier, Folke Brinkmann, Cordula Koerner-Rettberg, Sivagurunathan Sutharsan, Uwe Mellies, Christian Taube, Matthias Welsner

**Affiliations:** ^1^ Paediatric Pulmonology and Sleep Medicine, Cystic Fibrosis Center, Children’s Hospital, University of Duisburg-Essen, Essen, Germany; ^2^ Institute of Human Nutrition and Food Science, Christian-Albrechts-Universität zu Kiel, Kiel, Germany; ^3^ Department of Pediatric Pulmonology, University Children’s Hospital, Ruhr University Bochum, Bochum, Germany; ^4^ Children’s Hospital, Marienhospital Wesel, Wesel, Germany; ^5^ Department of Pulmonary Medicine, University Hospital Essen - Ruhrlandklinik, Adult Cystic Fibrosis Center, University of Duisburg-Essen, Essen, Germany

**Keywords:** cystic fibrosis, adult, habitual physical activity, elexacaftor/tezacaftor/ivacaftor, longitudinal effects, CFTR modulators

## Abstract

**Background:**

Habitual physical activity (PA) and exercise training are accepted as important aspects of care for people with cystic fibrosis (pwCF) to improve health-related measures of physical fitness, which in turn have a positive impact on quality of life and prognosis. In the last decade, effective CFTR modulator therapies have become a promising treatment for pwCF by targeting the underlying cause of CF. This highly effective therapy improves clinical outcomes and quality of life in people with specific CFTR mutations. Little is known about the longitudinal pattern of PA or the impact of the highly effective modulator therapy with Elexacaftor/Tezacaftor/Ivacaftor (ETI) on PA in adult pwCF. This study assessed the course of device-based PA measurement in adult pwCF and evaluated the effects of ETI on habitual physical activity in those who were eligible for ETI.

**Methods:**

Data from adult pwCF (aged ≥18 years) were analysed at baseline and follow-up, using identical assessments at both time points. Outcome parameters were PA in steps/day and the intensity of PA. The group that received ETI was treated for an average of 33 weeks and not for the entire duration of the period. The data were collected between 2021 and 2022, following the removal of absolute pandemic restrictions/lockdowns.

**Results:**

Follow-up duration was 5.6 years in pwCF with ETI (ETI group, *n* = 21) and 6.5 years in pwCF without ETI (non-ETI group, *n* = 6). From baseline to follow-up, pwCF treated with ETI had a significant increase in steps/day (+25%, *p* = 0.019) and a non-significant increase in moderate-to-vigorous intensity time (+5.6%, *p* = 0.352). Conversely, individuals in the non-ETI group showed a non-significant decrease in both steps/day −3.2%, *p* = 0.893) and moderate-to-vigorous intensity time (−25%, *p* = 0.207). The ETI group showed a significant decrease in percent predicted forced expiratory volume in 1 s (ppFEV_1_) and FEV_1_ z-score before the start of ETI treatment, both of which improved significantly after therapy initiation. Body weight and body mass index also improved significantly with ETI use.

**Conclusions:**

These data suggest that ETI treatment has a positive effect on habitual physical activity behavior in the adult pwCF studied.

## 1 Introduction

Cystic fibrosis (CF) is an autosomal recessive genetic disease that is caused by mutations in the gene responsible for the CF transmembrane conductance regulator (CFTR) (1, 2). This genetic defect is responsible for respiratory manifestations, exocrine pancreatic insufficiency, malabsorption and malnutrition, CF-related diabetes mellitus, reduced bone mass, osteoporosis, high hospitalization rates, impaired quality of life, and reduced life expectancy (1, 2). In recent years, improved therapeutic options and new drugs such as highly effective CFTR modulator therapy have been associated with a steady increase in life expectancy (1). The triple CFTR combination of Elexacaftor/Tezacaftor/Ivacaftor (ETI) is now available for up to 85% of all pwCF aged 12 years and older with at least one copy of the F508del mutation (3). Treatment with ETI results in a dramatic increase in lung function [percent predicted forced expiratory volume in 1 s (ppFEV_1_)], an improvement in nutritional status [body mass index (BMI)], a decrease in sweat chloride concentration, and fewer pulmonary exacerbations (4–8).

As well as medical treatment, numerous studies have shown that regular habitual physical activity and exercise have beneficial effects on exercise capacity, lung function, quality of life and prognosis in pwCF, regardless of disease severity (9, 10).

In line with the statement of the Copenhagen Consensus 2019, physical activity is considered in this study as an umbrella term encompassing both structured and unstructured forms of activity during leisure time, at home and at work (11). However, in this context, habitual physical activity can be described as physical activity that increases energy expenditure compared to rest. The intensity of physical activity is often described as light, moderate to vigorous or vigorous (12). Longitudinal changes in habitual physical activity in pwCF have not been widely studied. In children with CF, a higher level of self-reported habitual physical activity was found to correlate with a slower annual rate of decline in ppFEV1 (13). In addition, a higher level of moderate-to-vigorous intensity exercise was associated with better exercise capacity, slower decline in ppFEV_1_, and a lower hospitalization rate in adult pwCF (14, 15). Annual changes in habitual physical activity over period of more than 12 months recorded with questionnaires, accelerometry or both have not been investigated in adult pwCF to date. Outside of CF, studies in adult people with chronic obstructive pulmonary disease (pwCOPD) showed an annual decline in objectively measured habitual physical activity of between −451.0 steps and −393.7 steps/day over 5 and 3 years, respectively, without exercise intervention (16, 17).

The effects of CFTR modulator therapy on exercise capacity [e.g., peak oxygen uptake (VO_2_peak)] and on habitual physical activity are not clear. Some reports have found an improvement in exercise capacity (VO_2_peak, exercise time, distance walked in the 6 min walk test) (18–23) while others have found no or only minimal changes (24–26). Similar results can be seen for habitual physical activity. The effect of ETI treatment on habitual physical activity has only been investigated in one study. In the small group of three adolescents with pwCF studied, one individual showed a 17.1% increase in habitual physical activity time six weeks after starting ETI treatment, while another showed a 32.8 min decrease in daily PA (22).

The objectives of the present study were: (a) to investigate the longitudinal changes in habitual physical activity and time spent in different habitual physical activity intensities in pwCF treated with and without ETI; and (b) to determine the effects of ETI treatment on changes in habitual physical activity. We hypothesize that ETI will have beneficial effects on habitual physical activity levels and will improve habitual physical activity (steps/day) and the time spent in different habitual physical activity intensities, especially moderate-to-vigorous intensity activity.

## 2 Material and methods

### 2.1 Study design and participants

The present study is part of the CFmobil project, a partially supervised exercise program for pwCF aged ≥6 years, which has been described in detail elsewhere (27, 28). Briefly, eligible participants for the present study and statistical analysis were pwCF aged ≥18 years from the University Medicine Essen (Ruhrlandklinik, Essen, Children's Hospital, Bochum and Children's Hospital, Essen, Germany) who had already participated in the first part of CFmobil project. For the current analysis, only adult pwCF with or without ETI treatment who were willing to participate in follow-up were eligible.

All participants had a confirmed diagnosis of CF based on two defining mutations in the CFTR gene and gave written informed consent. Ethical approval was obtained from the ethics committee of the University Hospital Essen (14-6117-BO) and Bochum (15-53114), and is registered at clinicaltrials.gov (NCT03518697).

The trial was initially designed to evaluate the effects of a partially supervised exercise program on aspects of exercise capacity, habitual physical activity, quality of life and lung function. Following the introduction of ETI therapy, the study protocol was amended to include assessment of habitual physical activity over time and the effect of ETI treatment on habitual physical activity. Long-term effects on clinical outcome parameters (ppFEV_1_, FEV_1_ z-score, body weight and BMI) were also recorded. There was no overlap in time between the CFmobil exercise intervention and the start of the ETI treatment.

### 2.2 Treatment

ETI treatment was initiated after approval by the European Medicines Agency in 2020 and was given to those eligible for treatment (29). Some participants received ETI treatment as part of pre-registration studies, which explains the timing and duration of treatment before ETI was approved in Germany or Europe. The average duration of treatment with ETI in the ETI group was 33 weeks.

### 2.3 Measurements

**Figure 1:**
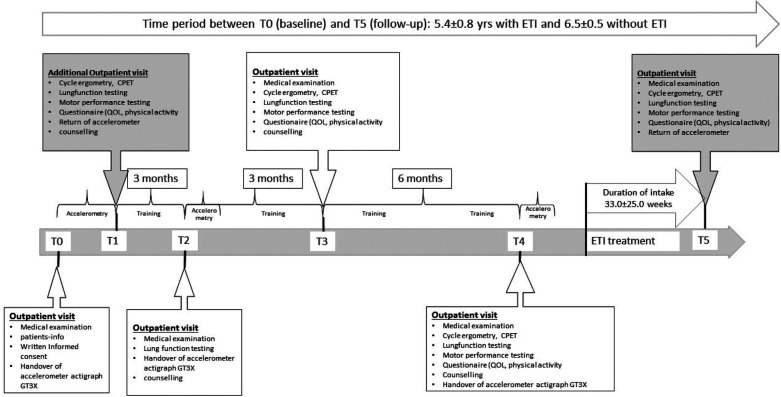
Overview of the CFmobil project, including details of the clinical examinations and different assessments over time. CPET, cardiopulmonary exercise testing; ETI, elexacaftor/tezacaftor/ivacaftor; QOL, quality of life; T1/T3/T4, physical fitness testing and counselling; T5, follow-up testing.

All participants underwent the same clinical assessments as at the start of the CFmobil project (shown in Figure 1). Baseline data (T0) were collected between 2014 and 2018, and follow-up data (T5) were collected between 2021 and 2022. In this study, habitual physical activity (steps/day and intensity) and the clinical outcome parameters ppFEV_1_, FEV_1_ z-score, weight and BMI were recorded and statistically analyzed. Measurements of clinical parameters were performed in the ETI-treated group at three different time points. These were at baseline, before the start of ETI treatment and at the end of follow-up.

### 2.4 Anthropometric characteristics and lung function

Body weight was measured using an electronic flat scale (Seca 861; Seca, Hamburg, Germany) and height was measured to the nearest 0.1 cm using a telescopic measuring rod (Seca 202; Seca, Hamburg, Germany). BMI was calculated as weight divided by the square of height (kg/m^2^). The ppFEV_1_ was assessed according to the guidelines of the American Thoracic Society (30) using standard spirometric techniques (JAEGER MasterScreen Body; CareFusion, Höchberg, Germany) and expressed as a percentage of the predicted value. FEV z-scores were calculated according to the Global Lung Initiative (31).

### 2.5 Physical activity

Habitual physical activity was recorded with a wActiSleep-BT Monitor (Actigraph Corp., Pensacola, FL, USA) over a period of four weeks at baseline and two weeks at follow-up, including one weekend. Only the first two weeks of baseline were used to compare habitual physical activity measurements at the different time points. Habitual physical activity-related parameters were steps/day and habitual physical activity intensities expressed as metabolic equivalents (METs: sedentary-to-light <3 METs min/day, moderate-to-vigorous intensity 3–5.9 METs min/day, and vigorous ≥6 METs min/day) (32, 33). The different intensity ranges of habitual physical activity are based on the cut points of Freedson et al. (34).

In addition, physical activity and sedentary behavior guidelines were considered to determine whether and to what extent participants met guideline recommendations for both time, steps and habitual physical activity intensity. These guidelines recommend a minimum of 150 min/week of moderate-to-vigorous physical activity (a minimum of 30 min on 5 days per week, which can be accumulated from bouts of 10 min or more towards the 30 min minimum) or 75 min of vigorous physical activity throughout the week (35–38). Expressed as habitual physical activity in steps per day, between 7,500 and 9,999 steps/day is considered as a physically active lifestyle, between 5,000 and 7,499 steps per day is considered a low active lifestyle, and less than 5,000 steps per day is considered a sedentary lifestyle (39).

### 2.6 Statistics

Continuous variables are presented as mean ± standard deviation with 95% confidence intervals. In a first step, the data were tested for a normal distribution by using the Shapiro–Wilk test. If the statistical analysis indicates that the data do not follow a normal distribution, all subsequent analyses were performed using non-parametric analysis methods. The following variables were included in the statistical analysis from baseline to follow-up: habitual physical activity (steps/day, and physical activity intensity), and clinical outcomes ppFEV_1_, FEV_1_ z-scores, body weight, and BMI. Changes over time were calculated using a Wilcoxon's signed rank test. In a second step, groups were analyzed separately for statistically significant changes over time. A Kruskal–Wallis test was used to identify differences between the groups with and without ETI therapy at baseline and follow-up.

A Friedman two-way analysis of variance (ANOVA) with rank test was performed within the ETI group to assess differences from baseline to follow-up, and *post hoc* tests were performed if the test result was significant. In addition, correlations between habitual physical activity, intensity of habitual physical activity, body weight, BMI, ppFEV_1_ and FEV_1_-z score were analyzed using Spearman correlation coefficients, and Cohen's d was calculated for the effects of ETI treatment on habitual physical activity and on the amount of moderate-to-severe intensity habitual physical activity. According to Cohen, effect sizes were categorized as small (|*d*| = 0.2), moderate (|*d*| = 0.5) or large (|*d*| = 0.8) (40).

Statistical significance was defined as *p* ≤ 0.05 level for all statistical tests. Statistical analyses were performed using SPSS 28.0 (IBM Corp. Version 28.0. Armonk, NY, USA).

## 3 Results

### 3.1 Anthropometric characteristics and clinical outcomes with and without ETI therapy

A total of 88 children, adolescents and adult pwCF were included in the CFmobil exercise program at baseline (41, 42). Complete follow-up was completed by 27 adult pwCF (67% males; age 18–50 years) of whom 21 received ETI therapy (shown in Table 1). Of six pwCF not treated with ETI, two refused treatment and four were not eligible due to their CFTR mutation.

**Table 1:** Demographic data and clinical outcome parameters of the ETI group from baseline to initiation of ETI and at follow-up and of the non-ETI group from baseline to follow-up.

	ETI group (*n* = 21)			non-ETI group (*n* = 6)		Between-group comparison *p*-value		Within-group comparison *p*-value	
	Baseline	Start of ETI	Follow-up	Baseline	Follow-up	Baseline	Follow-up	ETI group	non-ETI group
Male, *n* (%)	14 (67)			4 (67)					
del F508 homozygous, *n* (%)	13 (62)			4 (67)					
del F508 heterozygous (in %)	6 (28)			1 (17)					
Pancreatic insufficiency, *n* (%)	17 (80)			6 (100)					
*Pseudomonas aeruginosa*, *n* (%)	11 (52)			2 (33)					
Time between clinical assessments (years)	5.4 ± 0.8 (5.0/5.7)			6.5 ± 0.5 (6.1/7.0)		**0**.**001**			
Duration of ETI therapy (weeks)			33 ± 25 (21.0/44.0)			** **			
Age (years)	25.9 ± 7.4 (22.5/29.2)		31.0 ± 7.6 (27.5/34.4)^†††^	24.0 ± 7.9 (15.7/32.3)	30.3 ± 7.7 (22.2/38.4)^†^	0.316	0.860	**< 0.001**	**0**.**023**
Height (cm)	171.2 ± 8.8 (167.0/175.2)			175.0 ± 12.0 (162.4/187.6)	–				
Weight (kg)	58.7 ± 11.6 (53.7/64.0)	59.4 ± 11.4 (53.7/65.0)^###^	67.1 ± 11.9 (61.7/72.6)^†††^	60.0 ± 7.2 (54.0, 66.0)	62.3 ± 7.3 (56.2/68.4)	0.484	.502	**0.001**	0.150
BMI (kg/m^2^)	19.9 ± 2.8 (18.8 21.2)	20.5 ± 3.0 (19.0/22.0)^###^	22.8 ± 2.6 (21.6/23.9)^†††^	19.7 ± 1.2 (18.7/20.7)	20.5 ± 1.7 (19.1/22.0)	0.521	0.122	**< 0.001**	0.075
ppFEV_1_	51.9 ± 16.5 (44.4/59.5)	43.0 ± 16.1 (35.0, 51.6)**^,^^###^	53.1 ± 17.9 (45.0/61.3)	68.5 ± 21.6 (60.0/100.7)	63.0 ± 26.9 (41.6/88.7)	**0**.**004**	0.381	**< 0.001**	0.123
FEV_1_ z-score	−3.92 ± 1.3 (−4.5/−3.4)	−4.54 ± 1.2 (−5.12/−3.97)*^,^^###^	−3.0 ± 1.9 (−3.9/−2.1)^†^	−1.89 ± 1.75 (−3.73/−0.6)	−2.70 ± 1.68 (−4.46/0.94)	**0**.**012**	0.771	**< 0.001**	0.401

Values are number of patients (%) or mean ± standard deviation (95% confidence interval). BMI, body mass index; ETI, elexacaftor/tezacaftor/ivacaftor; ppFEV_1_, percent predicted forced expiratory volume in 1 s. Baseline, assessment of habitual physical activity; clinical assessment and testing; Follow-up, clinical assessment and testing between groups. Statistical testing: Kruskal–Wallis test (between groups); Mann–Whitney *U*-test (within groups); Friedman two-way analysis of variance by ranks test in the ETI group to compare the changes over time; Chi-Quadrat (2), *χ*² (*post hoc* tests).

* Baseline vs. initiation of ETI: *p* < 0.05.

** Baseline vs. initiation of ETI: *p* < 0.01.

^#^ Initiation of ETI vs. follow-up: *p* < 0.05.

^###^ Initiation of ETI vs. follow-up: *p* < 0.001.

^†^ Baseline vs. follow-up: *p* < 0.05.

^†††^ Baseline vs. follow-up: *p* < 0.001.

Bold type indicates a significant result.

The mean time between baseline assessment and follow up was 5.6 ± 0.8 years in the ETI group and 6.5 ± 0.5 years in the non-ETI group (*Z* = –3.005, *p* = 0.001). The mean duration of treatment with ETI was 33 ± 25 weeks (shown in Figure 1) and the mean time between baseline assessment and initiation of ETI was 4.9 ± 0.7 years.

The number of steps per day and the intensity of habitual physical activity were recorded in the ETI and non-ETI groups at baseline and at follow-up. In the ETI group, the HPA was not assessed prior to the initiation of ETI treatment.

Out of the total of *n* = 27 pwCF, *n* = 16 participated in the CFmobil project, with *n* = 14 from the ETI group and *n* = 2 from the non-ETI group. Overall, *n* = 9 pwCF had not received a CFTR modulator prior to ETI initiation, whereas *n* = 18 pwCF had been treated with a mono or dual CFTR modulator prior to ETI treatment.

### 3.2 Clinical outcomes

**Figure 2:**
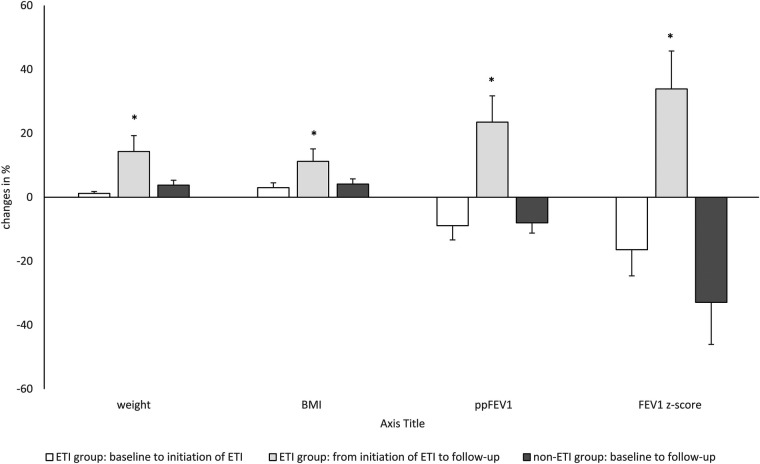
Longitudinal changes (in %) of clinical parameters (weight, BMI, body mass index), and lung function over time with ETI treatment of 33 weeks in the ETI group and without ETI treatment in the non-ETI group. ETI, elexacaftor/tezacaftor/ivacaftor; ppFEV1, percent predicted forced expiratory volume in 1 s; ETI, elexacaftor/tezacaftor/ivacaftor. Kruskal–Willis Test *p < 0.05.

Change in clinical parameters and lung function over time are shown in Table 1. At baseline, the ETI group had a significantly lower ppFEV_1_ and FEV_1_ z-score compared with the non-ETI group, whereas body weight and BMI were similar shown in (Tables 1, 2). In the non-ETI group, there was a non-significant deterioration of the ppFEV_1_ and the FEV_1_ z-score from the baseline assessment to the follow-up measurement Body weight and BMI improved slightly over time but changes did not achieve statistical significance (shown in Figure 2).

**Table 2:** Steps and intensity of habitual physical activity over time in the ETI group and the non-ETI group from baseline to follow-up.

	ETI group (*n* = 21)		non-ETI group (*n* = 6)		Between-group comparison *p*-value		Within-group comparison *p*-value	
	Baseline	Follow-up	Baseline	Follow-up	Baseline	Follow-up	ETI group	non-ETI group
Steps (number/day)	8,798 ± 3,176 (7,352/10,243)	10,997 ± 3,468 (9,354/12,600)*	11,530 ± 3,636 (8,490/14,569)	11,164 ± 3,724 (8,716/14,908)	**0** **.** **042**	0.785	**0**.**019**	0.893
Total change (steps/day)		2,131.3 ± 3,268.7 (556.4/3,707.3)		−305.7 ± 2,628.1 (−2,351.7, 1,740.3)			** **	** **
Annual change in steps/day		414.0 ± 699.5 (76.9/751.2)		−86.8 ± 401.0 (457.6/284.1)	0.060	** **	** **	** **
Sedentary-to-light intensity HPA (<3 METs), in min/day	1,218.5 ± 133.9 (1,149.6/1,287.4)	1,170.6 ± 117.6 (1,115.7/1,287.4)	1,254.1 ± 197.4 (763.8/1,744.4)	1,103.0 ± 136.8 (885.4/1,320.6)	0.491	0.278	0.543	0.416
Moderate-to-vigorous intensity HPA (3–5.9 METs), in min/day	106.3 ± 90.6 (60.2/140.3)	112.1 ± 74.1 (74.4/143.8)	123.0 ± 101.0 (17.0/229.0)	92.2 ± 36.8 (46.5/137.9)	0.466	0.946	0.352	0.207
Vigorous HPA (≥6 METs), in min/day	16.7 ± 18.7 (6.5/27.7)	13.6 ± 18.6 (8.2, 22.3)	19.6 ± 20.0 (2.9/36.4)	18.3 ± 18.3 (−0.9/7.6)	0.767	0.407	0.363	0.465

Values are mean ± standard deviation (95% confidence interval).

Baseline, assessment of habitual physical activity and clinical parameters; Follow-up, assessment of habitual physical activity and clinical parameters. Statistical testing: Kruskal–Wallis test (between groups); Mann–Whitney *U*-test (within groups); Friedman two-way analysis of variance by ranks test to compare the changes over time; Chi-Quadrat (2), *χ*² (*post hoc* tests).

HPA, habitual physical activity; ETI, elexacaftor/tezacaftor/ivacaftor; METS, metabolic equivalents.

Bold type indicates a significant result.

In the ETI group, there was a significant improvement in all clinical outcomes (body weight, BMI, ppFEV_1_, and FEV_1_ z-score). Post hoc analysis showed significant decreases from baseline to the start of ETI treatment for ppFEV_1_ and FEV_1_ z-score. Body weight and BMI remained almost constant until the start of ETI treatment. During ETI treatment, significant improvements were seen in all clinical parameters (body weight, BMI, ppFEV_1_ and FEV_1_ z-score).

### 3.3 Habitual physical activity

The amount and intensity of habitual physical activity is detailed in Table 2. At baseline, the ETI group had significantly lower habitual physical activity in steps per day than the non-ETI group, whereas at follow-up the number of steps per day was comparable between the two groups.

**Figure 3:**
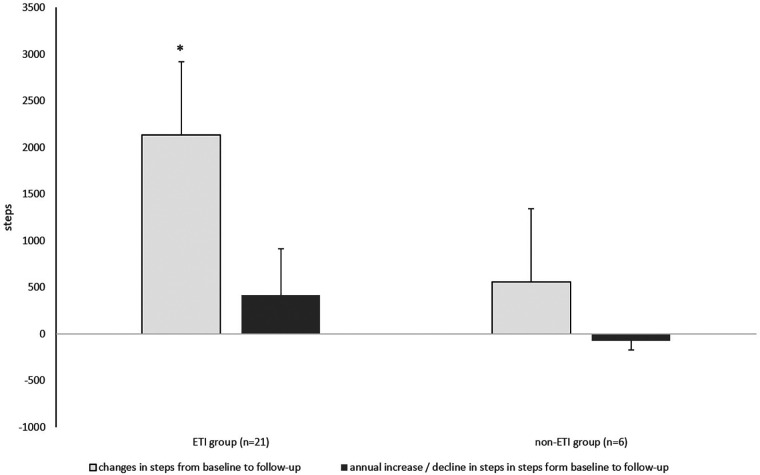
Absolute change and annual change in the number of steps with ETI treatment of 33 weeks in the ETI group and without ETI treatment the non-ETI group. ETI, elexacaftor/tezacaftor/ivacaftor. Kruskal–Willis Test *p < 0.05.

Over the observation period of 5.4 ± 0.8 years, habitual physical activity was significantly improved compared with baseline in the ETI group (by an average of 440 steps/day). However, it should be noted that the average duration of the ETI was 33 ± 25 weeks before the follow-up assessment. In contrast, pwCF in the non-ETI group showed a slight (but non-significant) reduction in habitual physical activity of −307.7 steps/day over the course of follow-up (mean 6.5 years), corresponding to a decrease in daily habitual physical activity of −86.8 steps/day (shown in Table 2 and in Figure 3). The between-group comparison for change in steps/day over time was of borderline statistical significance (*z* = −1.879, *p* = 0.060).

In both groups, the time spent in different habitual physical activity intensities changed slightly over time from baseline to follow-up (without becoming significant (shown in Table 2). In the non-ETI group, the time spent in moderate-to-vigorous intensity habitual physical activity decreased over time by −37.4 min/day, whereas in the ETI group there was a slight non-significant increase of 6.1 min/day (*z* = −0.719, *p* = 0.472, Cohen's *d* = 0.073). At baseline, 15% of participants in the ETI group spent <30 min/day in moderate-to-vigorous intensity habitual physical activity, whereas all participants in the non-ETI group achieved the 30 min/day threshold. At follow-up, the proportion of individuals in the ETI group who achieved the ≥30 min/day moderate-to-vigorous intensity habitual physical activity threshold decreased to 5%, whereas all participants in the non-ETI group exceeded this threshold (shown in Table 2 and Figure 3).

Over time, vigorous intensity habitual physical activity decreased in both groups, to a slightly greater extent in the ETI group than in the non-ETI group, and the ETI group spent slightly less time doing habitual physical activity at vigorous intensity than the non-ETI group.

Correlation coefficients between habitual physical activity (steps/day), habitual physical activity intensity and changes in clinical outcomes over time are shown in Table 3. In the ETI group, there was a significant association between changes in moderate-to-vigorous intensity in the ETI group and both ppFEV_1_ (rs = 0.620, *p* = 0.005) and FEV_1_ z-score (rs = 0.487, *p* = 0.035). In the non-ETI group, sedentary-to-light intensity correlated with FEV_1_ z-score (rs = 0.936, *p* = 0.037); there was also a positive but non-significant correlation between sedentary-to-light HPA and ppFEV_1_ in this group.

**Table 3:** Correlation coefficients between habitual physical activity, the intensity of habitual physical activity, and clinical outcomes (**A**) and between changes in habitual physical activity, the intensity of habitual physical activity, and clinical outcomes (**B**) in the ETI group and in the non-ETI group.

	ETI group (*n* = 21)								non-ETI group (*n* = 6)							
	Steps/day		SLHPA		MVHPA		VHPA		Steps/day		SLHPA		MVHPA		VHPA	
	*r*	*p*-value	*r*	*p*-value	*r*	*p*-value	*r*	*p*-value	*r*	*p*-value	*r*	*p*-value	*r*	*p*-value	*r*	*p*-value
A																
Baseline																
Body weight	0.267	0.255	−0.142	0.599	−0.083	0.729	0.142	0.549	−0.498	0.210	0.653	0.323	−0.360	0.381	0.484	0.224
BMI	0.481	**0**.**032**	−0.076	0.779	0.089	0.710	0.281	0.230	0.433	0.284	−0.297	0.628	0.628	0.096	−0.236	0.574
ppFEV_1_	0.177	0.457	0.148	0.533	−0.004	0.985	0.309	0.185	0.057	0.893	0.579	0.306	0.242	0.563	−0.272	0.514
FEV_1_ z-score	0.158	0.507	0.059	0.827	0.006	0.981	−0.382	0.096	0.040	0.924	0.539	0.349	0.332	0.422	−0.352	0.392
Follow-up																
Body weight	0.362	0.128	−0.241	0.320	0.289	0.479	−0.241	0.321	−0.841	**0**.**018**	−0.214	0.684	−0.030	0.950	−0.608	−0.200
BMI	0.482	0.068	−0.275	0.254	0.229	**0**.**038**	−0.318	0.185	−0.420	0.348	−0.605	0.203	0.049	0.918	−0.653	0.160
ppFEV_1_	0.575	**0**.**010**	−0.322	0.179	0.322	0.179	−0.394	0.095	−0.122	0.795	−0.456	0.363	−0.008	0.986	−0.535	0.274
FEV_1_ z-score	0.205	0.400	−0.001	0.997	0.065	0.793	0.316	0.187	−0.090	0.847	−0.691	0.129	0.121	0.797	−0.724	0.104
B																
Body weight	0.333	0.163	0.118	0.674	0.376	0.113	−0.135	0.581	0.234	0.614	−0.455	0.545	−0.001	0.998	−0.032	0.952
BMI	0.312	0.194	0.064	0.820	0.401	0.089	−0.102	0.678	0.130	0.780	−0.480	0.520	−0.128	0.785	0.039	0.941
ppFEV_1_	0.352	0.139	0.457	0.086	0.620	**0**.**005**	−0.027	0.913	−0.481	0.275	0.920	0.080	−0.168	0.719	0.101	0.859
FEV_1_ z-score	−0.040	0.871	0.077	0.785	0.487	**0**.**035**	−0.007	0.976	−0.323	0.480	0.936	**0**.**037**	0.078	0.868	−0.022	0.967

BMI, body mass index; ETI, elexacaftor/tezacaftor/ivacaftor; ppFEV_1_, percent predicted forced expiratory volume in 1 s; SLPHA, sedentary-to-light habitual physical activity; MVHPA, moderate-to-vigorous habitual physical activity; VVHPA, very vigorous habitual physical activity. Baseline, assessment of habitual physical activity; clinical assessment and testing; Follow-up, clinical assessment and testing between groups. *r,* Pearson's correlation coefficient.

Bold type indicates a significant result.

## 4 Discussion

Our data show a significant increase in habitual physical activity, measured in steps/day, and a slight increase in moderate-to-vigorous intensity over a period of 5.6 years in pwCF treated with ETI for 33 ± 25 weeks. In the non-ETI group, there was a slight decrease habitual physical activity and in moderate-to-vigorous intensity habitual physical activity over 6.5 years. However, it should be noted that habitual physical activity was only recorded at the baseline and end of the observation period (follow-up), but not before the start of ETI treatment.

To date only a few studies have objectively measured longitudinal changes in the physical activity behavior of adult pwCF. Most of these investigated the effects of exercise interventions (e.g., partially supervised exercise programs consisting of aerobic endurance and/or strength training) on physical activity behavior. All reported an increase in objectively measured steps/day or different intensity levels of the habitual physical activity after the intervention, and these beneficial effects persisted for up to 12 months after the intervention (43–45).

Studies of pwCF who participated in a partially supervised exercise intervention showed a more persistent positive effect on physical fitness parameters (43, 44). During the longitudinal observation period from baseline to follow-up in our study, some participants also participated in CFmobil, a partially supervised 12-month exercise intervention (41, 42).

However, positive health effects of a 6-month, partially supervised home exercise programme were seen up to 24 months after the end of the intervention. In contrast, in our own previously published study, we were not able to show these long-term effects of exercise on physical fitness (27). The time between participation of some pwCF in CFmobil, and follow-up was on average 4.5 years. It may be feasible that the training programme led to some pwCF continuing to participate in physical activity, which could explain the higher step counts at follow-up in combination with the use of ETI. As there are no longitudinal studies of the effects of exercise interventions over more than 24 months after cessation in pwCF, with and without ETI treatment, it is difficult to draw conclusions regarding the effects of the CFmobil training intervention on habitual PA.

However, the improvement in daily steps seen in the ETI group (about 25%) was higher than in a study by Causer et al., who examined at the effects of ETI treatment on exercise capacity (VO_2_peak) and habitual physical activity six weeks after starting ETI treatment (22). Of the three pwCF in this study, one showed a 17% increase in steps/day and another showed a 32% decrease in steps/day. Device-based data were not available for one participant due to poor adherence. This suggests that despite the short-term positive effects of ETI treatment, such as a signifcant increase in ppFEV1, other factors may also be important in motivating pwCF to engage in regular physical activity and exercise. This may indicate that behavioural changes in physical activity require a longer treatment period to become effective in terms of increased physical activity. However, due to the small number and young age of the people studied in the study by Causer et al, a comparison with our results is limited.

Some of our participants received a mono or dual CFTR modulator treatment prior to initiation of ETI therapy. Many studies have shown that pwCF treated with a CFTR modulator had better-preserved lung function and improved nutritional status (46, 47). Few studies have investigated the effects of CFTR modulators on physical activity and fitness parameters, and it is still unclear whether treatment with CFTR modulators has a beneficial effect on both (8, 18, 19, 21–24, 26, 48). Thus, it cannot be excluded that the use of mono or dual CFTR modulators may have had a positive impact in some pwCF on the number of steps/day and time spent in moderate-to-vigorous exercise even before ETI therapy. In the present study, there was a significant correlation between the number of steps/day at baseline and changes in ppFEV_1_ and habitual physical activity at moderate-to-vigorous intensity in the ETI group but not in the non-ETI group (Table 3). The time spent doing moderate-to-high intensity habitual physical activity in the ETI group increased by 5.6%. This suggests that ppFEV_1_ or disease severity may be related to both steps/day and habitual physical activity intensity, and their changes over time (9). The improvement in ppFEV_1_ that occurred during ETI treatment appears to have a positive effect on various aspects of daily life (more energy, easier task completion, better sleep quality), which reduces the negative sensations associated with habitual physical activity in everyday life (e.g., shortness of breath and coughing). This, combined with less time spent on chest physiotherapy because of reduced sputum production, might increase the motivation to spend more time on moderate-to-vigorous habitual physical activity (49, 50).

Individuals who were not taking ETI in our analysis reduced both the overall level and intensity of habitual physical activity over 6.5 years; the annual decrease was 86.8 steps/day. This decrease in habitual physical activity in the non-ETI group is lower than that reported in a study by Sievi et al. in pwCOPD (17). The natural progression of the disease, deconditioning and physical inactivity appeared to be more pronounced in pwCOPD, which may be attributed to a higher age (COPD: mean age 64.0 years vs. pwCF mean age 24.0 years) and more disease progression. Although our pwCF were younger, the possibility that the decrease in the number of steps per year is due to natural disease progression cannot be excluded (14, 15).

The sample size in our cohort was small, which may have biased the results in terms of number of steps. For example, two of the pwCF without ETI treatment who had participated in previous tests and the CFmobil exercise program reported that the exercise tests (bicycle ergometry and motor performance tests, regular counselling, (Figure 1) motivated them to increase their physical activity (habitual and otherwise). This suggests that regular monitoring of pwCF might be necessary to encourage or sustain motivation to participate in physical activity and exercise (51–53).

It should also be noted that factors other than lung function may affect exercise behavior in pwCF. Specifically, personal factors (motivation, energy level, time, illness, pain, dyspnea, confidence in exercising), environmental factors (weather, season, accessibility of training sites) and socio-demographic characteristics (e.g., education, study or occupation) may support or prevent participation in habitual physical activity (51, 52). The individual relevance of these factors may change over time, for example with disease severity and age, and may prevent or facilitate the realization of habitual physical activity, especially in the non-ETI group. The present study did not ask about the reasons for participation or non-participation in habitual physical activity. The ETI group had a significantly lower lung function at baseline than the non-ETI group, and it is likely that each barrier had a greater or lesser impact on participation in physical activity.

Physical activity guidelines recommend that people should take more than 7,500 steps/day and do at least 150–300 min of moderate-to-vigorous aerobic physical activity (3–5.9 METs) per week (35–38). On average, our participants met these recommendations at both baseline and follow-up. The number of steps/day achieved at both time assessments was comparable to other studies of adult pwCF (54–56). However, when focusing on individual steps/day in both groups at baseline (without ETI treatment), we found that half of the participants who went on to start ETI therapy had a low-activity lifestyle (5,000–7,499 steps/day) and 15% of those who did not start ETI treatment group took fewer than 7,500 steps/day. This habitual physical activity behavior suggests a physically inactive daily routine and possibly deconditioning in some of the pwCF, especially those with reduced lung function in the ETI group. It is interesting to note that at the end of the follow-up period, 79% of participants in the ETI group and 87.5% in the non-ETI group exceeded the recommended threshold of 7,500 steps. Twelve participants from the ETI group and two from the non-ETI group had already participated in the partially controlled stress intervention CFmobil. The increase in steps at the end of the observation period could also be due to a sustained effect of the CFmobil project, in addition to the positive effects of the ETI treatment. This was not investigated in the present study and can therefore only be hypothesized.

Increased habitual physical activity in pwCF is associated with better overall health, as it is in healthy adults (10, 35, 36, 57). This is important because studies have shown that overweight and obesity are on the rise in adult pwCF, especially during ETI therapy. Furthermore, treatment with ETI might increase blood pressure, which increases the risk of cardiovascular disease (58–60). Regular physical activity has been shown to be beneficial in the prevention and management of cardiovascular and metabolic conditions such as heart disease, stroke or diabetes. In addition, as well as being beneficial for the prevention of non-communicable diseases (e.g., hypertension, cardiovascular disease, overweight/obesity) physical activity can also improve mental health, quality of life and well-being (18, 37, 53). In this context, regular habitual physical activity and exercise will be of great importance in pwCF in the future because non-communicable diseases will increase due to ETI therapy and improved life expectancy.

In general, a physically inactive lifestyle is a common problem among pwCF (10), as seen for some of the pwCF in our study. Also in terms of non-communicable disease prevention, a higher intensity of habitual physical activity in pwCF could be beneficial, as a recently published retrospective study has reported in the UK Biobank cohort (61). It was shown that just 15–20 min of vigorous exercise per week (or 2 sessions per day of up to 2 min) was associated with a 35% reduction in cardiovascular mortality and a lower incidence of cardiovascular disease and cancer. The dose-response relationship between exercise (e.g., amount, duration, frequency, intensity) and improvements in health and physical fitness in pwCF with and without CFTR modulator treatment has not been studied (10). Further research in CF should therefore investigate the dose-response relationship between of habitual physical activity in different exercise interventions on physical performance and health benefits, both in general and in the context of ETI treatment.

As we expected, there was a significant improvement in clinical outcome parameters, including BMI, ppFEV_1_ and FEV_1_ z-scores, after the initiation of ETI, which is consistent with recently published studies (4–7). In the non-ETI group, body weight and BMI remained stable over time while lung function declined longitudinally, as seen in previous studies (4–8).

When discussing the results of this longitudinal study, some limitations need to be considered. Follow-up examinations were performed from 2021 to 2022 in the same way as the initial examinations, and only data from patients aged ≥18 years at baseline were used for the present analysis. Prior to the start of ETI treatment, only clinical outcome parameters were recorded, but not habitual physical activity. Habitual physical activity data were only collected at the start of ETI therapy and follow-up. Therefore, conclusions about longitudinal changes in habitual physical activity prior to the start of treatment with ETI are limited. The duration of ETI therapy in our pwCF varied between 13 and 118 weeks, and effects of treatment on body weight and lung function can be detected over a short period of drug use (4–8). However, it is likely that the effects of ETI treatment on physical activity behavior will take longer to become evident, and the effects may be greater with longer use. The clinical assessments at follow-up took place after the COVID-19 pandemic and there were no pandemic-related restrictions. However, it is possible that there may be a small effect on habitual physical activity due to individual social distancing restrictions leading to a reduction in social activities and habitual physical activity. We did not take this into account in our study. Finally, only a small subset of the study population was not treated with ETI because only a minority were not eligible for ETI treatment, which may have biased the results for this group.

## 5 Conclusion

This longitudinal study highlights the efficacy and beneficial effects of 33 weeks of ETI treatment over a total of 5.4 years on habitual physical activity in a group of adult pwCF. Compared with the non-ETI group, ETI treatment slowed the decline or improved habitual physical activity levels. Without ETI treatment, a moderate annual decrease in habitual physical activity volume and intensity levels was observed. The treatment with ETI seemed to motivate the pwCF who took part in the study to increase the amount of physical activity they usually carried out. This could be an important consideration for the future in terms of preventing non-communicable diseases that may occur as a result of treatment with CFTR modulators and the increased life expectancy in pwCF. Further studies with larger samples are therefore needed to investigate the long-term changes in daily physical activity, in the presence or absence of CFTR modulator therapy.

## Data Availability

The data that support the findings of this study are not publicly available due to applicable data protection regulations, as they contain information that could compromise the privacy of research participants. Requests to access the datasets should be directed to WG, wolfgang.gruber@uk-essen.de.
